# Editorial: Post-transplant monitoring for allograft rejection

**DOI:** 10.3389/frtra.2025.1693261

**Published:** 2025-09-19

**Authors:** Reut Hod-Dvorai, Reginald Gohh

**Affiliations:** ^1^ Department of Pathology, SUNY Upstate Medical University, Syracuse, NY, United States; ^2^ Department of Medicine, Georgetown University, Washington, DC, United States

**Keywords:** post-transplant, monitoring, rejection, biomarkers, donor-derived cell free DNA, donor-specific antibodies, biopsy

**Editorial on the Research Topic**
Post-transplant monitoring for allograft rejection

Post-transplant monitoring for allograft rejection is critical for identifying and treating events that may cause graft damage and graft loss. It is especially important in cases of delayed graft function, switching or adjusting immunosuppression, monitoring rejection treatment response and checking for medical adherence. Post-transplant monitoring is a broad term that commonly includes the use of biopsies (surveillance or for cause), functional parameters (e.g., Creatinine) and more recently, biomarkers. The utilization of biomarkers in post-transplant monitoring for allograft rejection allows for enhanced personalized management of immunosuppression and early therapeutic intervention at the onset of graft damage. Ideally, these biomarkers would be sensitive, cost-effective and offer a non-invasive alternative to biopsies. There are three main categories of biomarkers of post-transplant monitoring: 1) immunological markers such as donor-specific antibodies against human leukocyte antigens (HLA) and antibodies against non-HLA targets, cytokine & chemokine levels, etc.; 2) donor-derived cell free DNA; 3) Gene expression markers measured in blood, urine or biopsy tissue. Utilizing a variety of biomarkers may provide a more comprehensive picture and allow for better assessment and management of post-transplant patients. This Research Topic features four manuscripts discussing the utilization of different biomarkers in post-transplant monitoring for thoracic and abdominal transplants.

Kattih and Aryal discuss the use of biomarkers such as anti-HLA donor-specific antibodies, donor-derived cell free DNA (dd-cfDNA), immune cell function (ICF) and next-generation sequencing of microorganisms to monitor graft dysfunction and distinguish between rejection and infection in the setting of lung transplantation. Philogene et al. share their perspective on current challenges in non-HLA antibody testing for post-transplant monitoring and argue that a systematic validation of non-HLA antibody panels and assays is required as a first step to standardize testing and assess the clinical impact of non-HLA antibodies on transplant outcome. Bahniwal et al. explore the utility of dd-cfDNA as a non-invasive approach in post-transplant monitoring of heart transplant recipients, focusing on how to manage cases in which there is a disagreement between biopsy and dd-cfDNA results. Finally, Radomsky et al. identify a core signature of cytokines and chemokines in endomyocardial biopsies (EMB) associated with rejection after heart transplantation. Interestingly, this EMB cytokine/chemokine pattern was distinct from the pattern observed in plasma samples, arguing for a local protein microenvironment associated with rejection.

As different indications lead to different post-transplant treatment plans, it is important to monitor and determine the causes for graft dysfunction. The papers in this Research Topic highlight the value of having multiple platforms to inform clinical decision-making and better care for transplant recipients.

